# Molecular evidence of Ebola Reston virus infection in Philippine bats

**DOI:** 10.1186/s12985-015-0331-3

**Published:** 2015-07-17

**Authors:** Sarah I. Jayme, Hume E. Field, Carol de Jong, Kevin J. Olival, Glenn Marsh, Anson M. Tagtag, Tom Hughes, Anthony C. Bucad, Jennifer Barr, Rachel R. Azul, Lilia M. Retes, Adam Foord, Meng Yu, Magdalena S. Cruz, Imelda J. Santos, Theresa Mundita S. Lim, Carolyn C. Benigno, Jonathan H. Epstein, Lin-Fa Wang, Peter Daszak, Scott H. Newman

**Affiliations:** Food and Agriculture Organization of the United Nations, Makati City, Philippines; Queensland Centre for Emerging Infectious Diseases, Department of Agriculture, Fisheries and Forestry, Brisbane, Australia; EcoHealth Alliance, New York, USA; CSIRO Australian Animal Health Laboratory, Geelong, Australia; Biodiversity Management Bureau, Department of Environment and Natural Resources, Quezon City, Philippines; Bureau of Animal Industries, Department of Agriculture, Quezon City, Philippines; Food and Agriculture Organization of the United Nations Regional Office for Asia and the Pacific (FAO RAP), Bangkok, Thailand; Program in Emerging Infectious Diseases, Duke-NUS Graduate Medical School, ᅟ, Singapore; Food and Agriculture Organization of the United Nations, Emergency Centre for Transboundary Animal Disease, Hanoi, Vietnam; Global Alliance for Rabies Control, Santa Rosa City, Philippines

**Keywords:** Reston, Ebolavirus, Filovirus, Philippine, Bat, Molecular, Serology

## Abstract

**Background:**

In 2008–09, evidence of Reston ebolavirus (RESTV) infection was found in domestic pigs and pig workers in the Philippines. With species of bats having been shown to be the cryptic reservoir of filoviruses elsewhere, the Philippine government, in conjunction with the Food and Agriculture Organization of the United Nations, assembled a multi-disciplinary and multi-institutional team to investigate Philippine bats as the possible reservoir of RESTV.

**Methods:**

The team undertook surveillance of bat populations at multiple locations during 2010 using both serology and molecular assays.

**Results:**

A total of 464 bats from 21 species were sampled. We found both molecular and serologic evidence of RESTV infection in multiple bat species. RNA was detected with quantitative PCR (qPCR) in oropharyngeal swabs taken from *Miniopterus schreibersii*, with three samples yielding a product on conventional hemi-nested PCR whose sequences differed from a Philippine pig isolate by a single nucleotide. Uncorroborated qPCR detections may indicate RESTV nucleic acid in several additional bat species (*M. australis*, *C. brachyotis* and *Ch. plicata*). We also detected anti-RESTV antibodies in three bats (*Acerodon jubatus*) using both Western blot and ELISA.

**Conclusions:**

The findings suggest that ebolavirus infection is taxonomically widespread in Philippine bats, but the evident low prevalence and low viral load warrants expanded surveillance to elaborate the findings, and more broadly, to determine the taxonomic and geographic occurrence of ebolaviruses in bats in the region.

## Background

Ebolaviruses were first described in 1976, aetiologically associated with outbreaks of human haemorrhagic fever in central and western Africa [1]. While outbreaks were sporadic, the high mortality rate of Ebolaviruses and the related Marburgviruses (family *Filoviridae*) demanded elaboration of their ecology. The origin of the viruses was cryptic [2, 3] and remained elusive until Leroy et al. [4] reported serological and molecular evidence of fruit bats as reservoirs of Ebola virus. Subsequent studies have revealed evidence of filovirus infection in multiple species of bats globally [5], including Africa [1, 6–8], Europe [9] and Asia [10, 11]. Reston virus (RESTV) was first described in 1989 when macaques imported from the Philippines to Reston, Virginia in the USA developed febrile, haemorrhagic disease, and asymptomatically infected several animal attendants working in the primate research facility [12, 13]. In 2008–09, RESTV was detected in domestic pigs and pig workers [14, 15] in the Philippines. In 2010, under the auspices of the Food and Agriculture Organization of the United Nations (FAO), we investigated Philippine bats as possible wildlife reservoirs of RESTV. Here we present the findings of this surveillance.

## Results

**Fig. 1:**
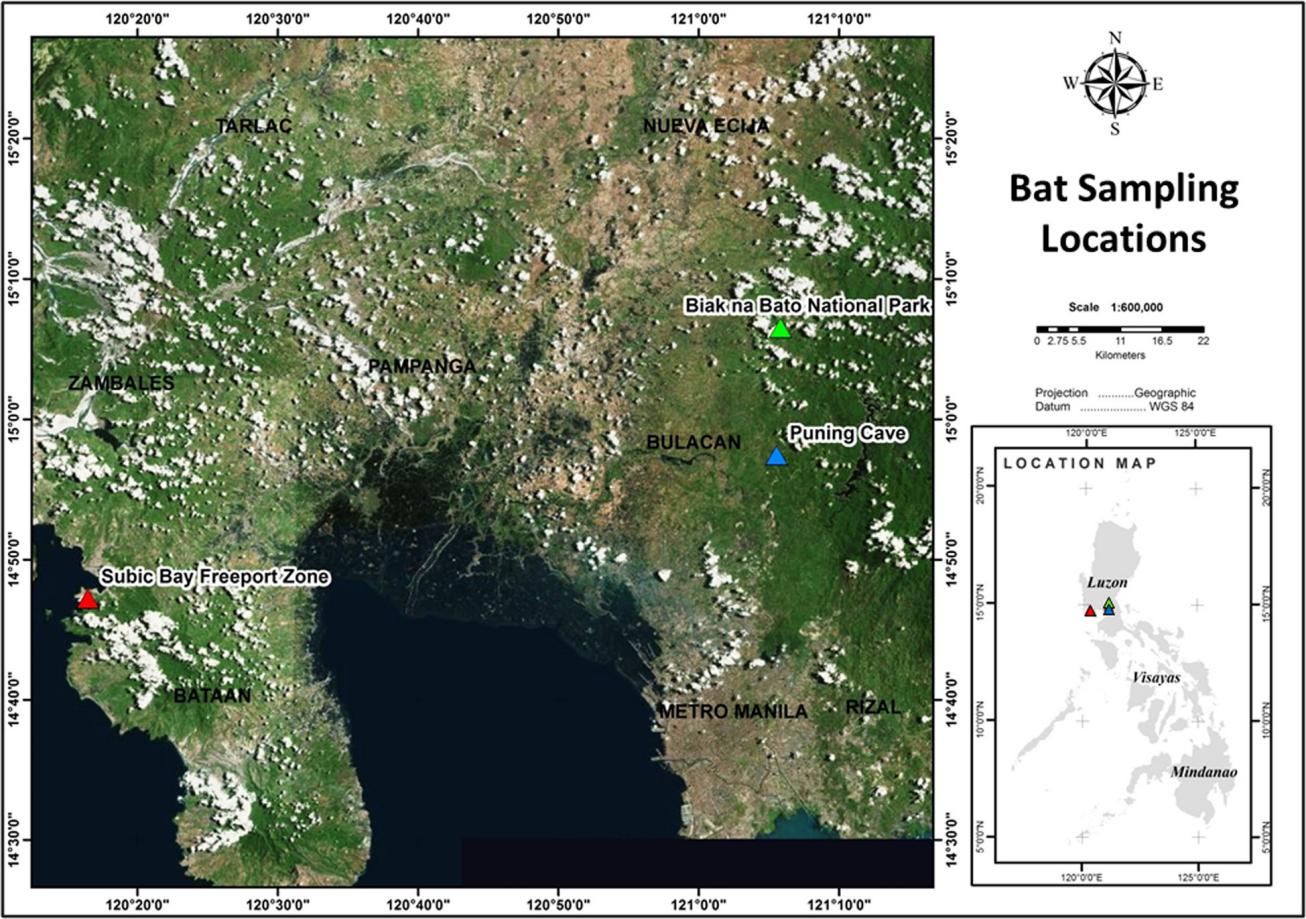
Bat sampling locations in Bulacan Province and Subic Bay Freeport Zone on the Philippine island of Luzon

A total of 464 bats were captured, comprising 403 bats from 19 species at Bulacan and 61 bats from two species at Subic Bay (Fig. 1) (Table 1). Bulacan yielded 351 serum samples and 739 swab samples (148 pools) suitable for testing: 299 oropharangeal swabs (60 pools), 248 rectal swabs (50 pools) and 192 urine swabs (38 pools). A complete suite of samples was not collected from all bats. Subic Bay yielded 61 serum samples and 183 swab samples suitable for testing: 61 oropharangeal swabs, 61 rectal swabs, 31 urogenital swabs and 30 urine samples.

**Table 1:** Details of 464 bats captured at two locations on the Philippine island of Luzon in 2010

Species	Count	Location
Fruit-bats		
Pteropodidae		
*Acerodon jubatus*	56	Subic Bay
*Cynopterus brachyotis*	37	Bulacan
*Eonycteris robusta*	11	Bulacan
*Eonycteris spalea*	1	Bulacan
*Ptenochirus jagori*	52	Bulacan
*Pteropus vampyrus*	5	Subic Bay
*Rousettus amplexicaudatus*	42	Bulacan
Insectivorous bats		
Mollosidae		
*Chaerephon plicata*	82	Bulacan
Hipposideridae		
*Hipposideros ater*	1	Bulacan
*Hipposideros diadema*	6	Bulacan
*Hipposideros pygmeus*	8	Bulacan
*Hipposideros sp.*	1	Bulacan
Vespertillionidae		
*Miniopterus australis*	70	Bulacan
*Miniopterus schreibersii*	44	Bulacan
*Murina cyclotis*	1	Bulacan
*Myotis horsfieldii*	5	Bulacan
*Tylonycteris robustula*	3	Bulacan
Rhinolophidae		
*Rhinolophus arcuatus*	31	Bulacan
*Rhinolophus philippinensis*	1	Bulacan
*Rhinolophus rufus*	6	Bulacan
*Rhinolophus virgo*	1	Bulacan
Total	464	

**Fig. 2:**
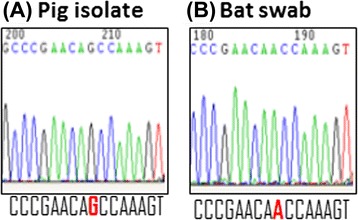
Comparison of sequencing trace files showing the 1-nt difference. **(a)** Sequence from the earlier Bulacan Farm A pig isolate; **(b)** Sequence from bat oropharangeal swab T69. Identical sequences were obtained from bat oropharangeal swabs T70 and T71 (not shown). The single nucleotide difference is highlighted in bold and red, which corresponds to nt residue 1,274 of the Reston ebolavirus isolate RESTV/Sus-wt/PHL/2009/09A Farm A (GenBank accession number JX477165.1)

**Fig. 3:**
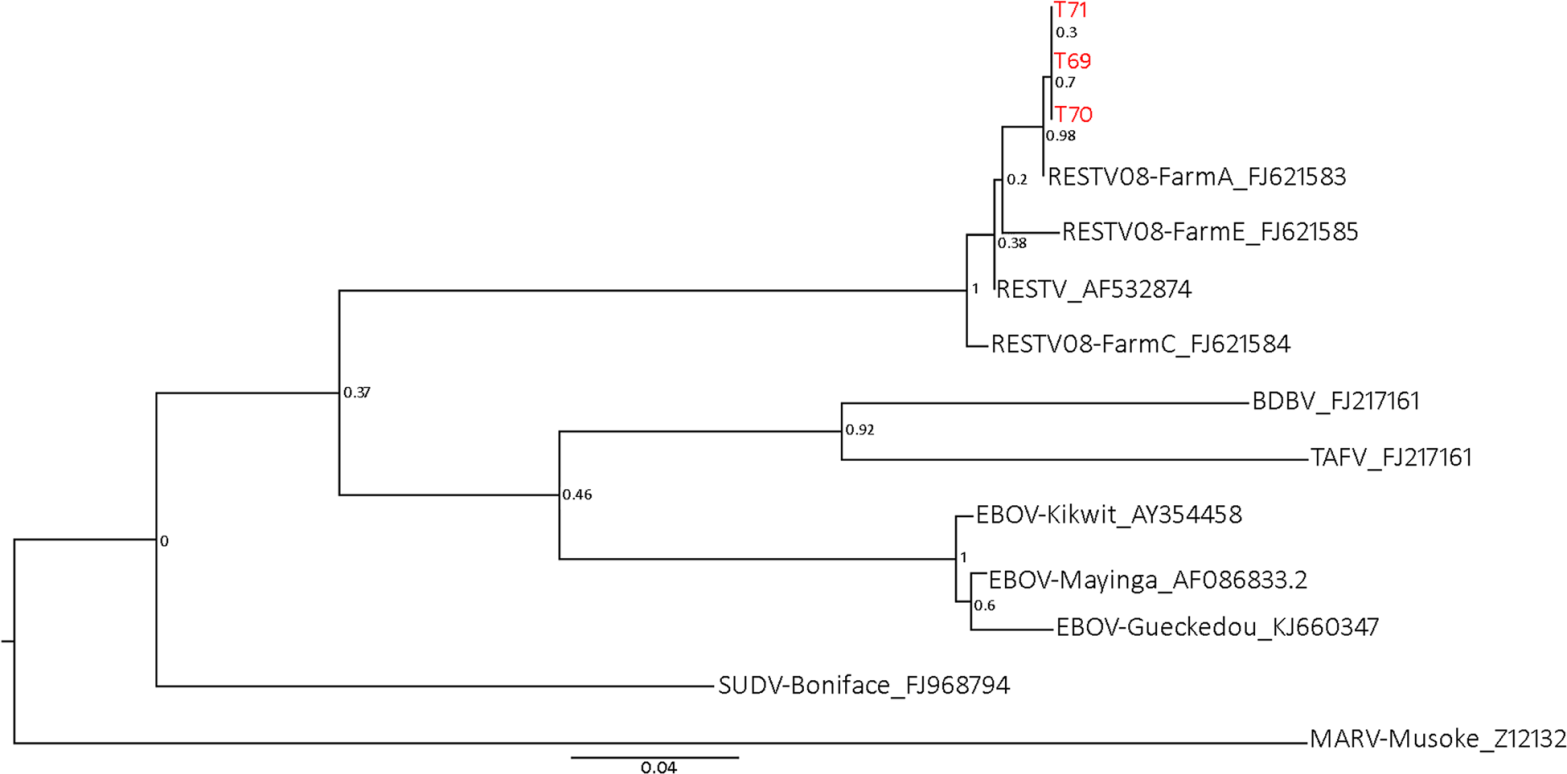
Phylogenetic analysis by maximum likelihood method, based on partial NP sequences (519 bp) obtained from hemi-nested PCR. Bat-derived RESTV sequence are shown in red

Of the Bulacan samples, all sera were negative on ELISA, and all rectal and urine swabs pools were negative for RESTV RNA on qPCR. Five oropharangeal swab pools returned potentially positive results on qPCR (Table 2). Each of the 25 component individual samples of the five pools was then tested individually. Three of these individual samples (from the same pool) yielded positive results (Table 2). All three samples were from *Miniopterus schreibersii* caught in the same cave on the same day. In the conventional PCR, all three samples yielded a product whose sequence differed by one nucleotide from a pig isolate sequence from Farm A [14] in Bulacan Province (Fig. 2). Likewise, in the phylogenetic analysis, the three bat-derived PCR product sequences are most related to the Reston isolate from Farm A (Fig. 3). Subsequent testing of 23 duplicate and five additional (*M. schreibserii*) oropharangeal swabs held by the PAHC laboratory in the qPCR yielded six samples with potentially positive results (four of which were *Miniopterus* species), including two of the three previously identified positives (Table 2). Conventional PCR was unable to generate a clean PCR product for direct sequencing of the PAHC duplicate samples because of the small sample volume and limited RNA present.

**Table 2:** qPCR results on original and archived PAHC duplicate oropharangeal swabs from five pools screening potentially positive^a^

Pool	Animal ID	Species	Location	Original sample pool C_t_	Original sample: individual C_t_	Duplicate sample: individual C_t_
2	U95	*C. brachyotis*	Puning Cave	42.0/ND		41.2/ND
	U96	*Pt. jagori*	Puning Cave			
	U97	*Pt. jagori*	Puning Cave			
	U98	*Pt. jagori*	Puning Cave			
	U99	*Pt. jagori*	Puning Cave			
3	R1	*M. australis*	Biak na Bato	43.0/ND		
	R2	*M. australis*	Biak na Bato			
	R3	*M. australis*	Biak na Bato			
	R4	*M. australis*	Biak na Bato			39.3/ND
	R5	*M. australis*	Biak na Bato			
4	T67	*M. australis*	Biak na Bato	40.6/41.9^b^		
	T68	*M. australis*	Biak na Bato			
	T69	*M. schreibersii* ^c^	Biak na Bato		33.6^d^	
	T70	*M. schreibersii* ^c^	Biak na Bato		37.7^d^	39.9/ND
	T71	*M. schreibersii* ^c^	Biak na Bato		32.9^d^	40.1/ND
	T72	*M. schreibersii* ^*e*^	Biak na Bato			
	T73	*M. schreibersii* ^e^	Biak na Bato			
	T74	*M. schreibersii* ^e^	Biak na Bato			40.5/ND
	T75	*M. schreibersii* ^e^	Biak na Bato			
	T76	*M. schreibersii* ^e^	Biak na Bato			
6	T56	*Ch. plicata*	Biak na Bato	39.7/40.1^b^		
	T57	*Ch. plicata*	Biak na Bato			
	T58	*Ch. plicata*	Biak na Bato			
	T59	*Ch. plicata*	Biak na Bato			
	T60	*M. schreibersii*	Biak na Bato			
12	U21	*M. australis*	Biak na Bato	40.2/ND		42.2/ND
	U22	*M. australis*	Biak na Bato			
	U23	*Ch. plicata*	Biak na Bato			
	U24	*Ch. plicata*	Biak na Bato			
	U25	*Ch. plicata*	Biak na Bato			

^a^All samples were tested in duplicate. Positive samples were confirmed in triplicate

^b^Pools 4 and 6 had repeatable results on the original pooled sample

^c^T69, T70 and T71 yielded a product on hemi-nested PCR whose sequence differed by one nucleotide from a pig isolate in Bulacan province

^d^Mean value of the duplicates

^e^Additional *M. schreibersii* samples from a pool which tested negative in the original round

**Fig. 4:**
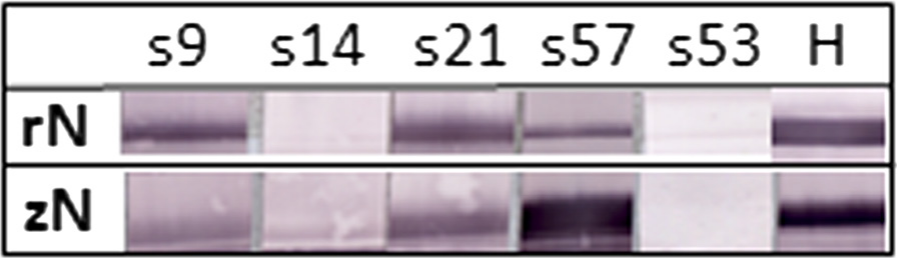
Western blot analysis. Recombinant nucleoproteins from RESTV (rN) and EBOV (zN) were used to probe for reactivity in four ELISA positive sera (s9, s21, s53 and s57) and one ELISA negative serum (s14). Anti-His tag monoclonal antibody (H) was used as a positive control

Of the Subic Bay samples, four sera were potentially positive on ELISA: three from *Acerodon jubatus* (s9, s21, s57), and one from *Pteropus vampyrus* (s53). Three (s9, s21, s57) were also positive on Western blot (Table 3). One sample (s57) showed a stronger response to EBOV than to RESTV antigen (Fig. 4). All samples and swabs were negative for RESTV RNA on qPCR.

**Table 3:** Positive serologic findings in 61 flying-foxes^a^ screened for anti-RESTV antibodies by ELISA and Western blot

Bat/sample ID	Species	Locality	ELISA	Western blot
s9	*A. jubatus*	Subic Bay	+	+
s21	*A. jubatus*	Subic Bay	+	+
s53	*P. vampyrus*	Tala	+	
s57	*A. jubatus*	Subic Bay	+	+^b^

^a^Comprising 56 *A. jubatus* and 5 *P. vampyrus* from two locations

^b^s57 showed a stronger reactivity to EBOV than to RESTV

## Discussion

We detected both serologic and molecular evidence of RESTV infection in Philippine bats. RESTV RNA in the oropharyngeal swab of three *Miniopterus schreibersii* clustered phylogenetically with the 2008 pig-derived sequences and the historic 1989 Philippine primate-derived sequence. Sequence from all three bats was identical, and aligned most closely with the 2008 pig isolate from a farm (Farm A) in Bulacan Province [14], less than 40 km from the bat sampling location. All sequenced products from bats had the single nucleotide change; all positive control and related material held at AAHL did not have the change. Limited variation is not surprising with an assay targeting a conserved region of the NP gene following recent introduction of infection a population. While the high C_t_ values from the qPCR indicate the assay is approaching the limits of detection with these samples, a number of factors support the veracity of the findings. At the laboratory level, the repeatability of positive findings using qPCR in both pooled and individual specimens, the repeatability of positive findings in archived duplicate specimens, the corroboration by conventional PCR, and the direct sequencing results. We detected RNA in archived duplicate samples of two of the three positive *M. schreibersii*; these duplicates were made in the Philippines, stored at the PAHC, and forwarded to AAHL and tested in a separate run 12 months after the first test. At the epidemiological level, the clustering of the positive samples in one species, in one cave, and in one sampling event is consistent with a current infection dynamic. The inability of the PAHC duplicate sample testing to fully corroborate the first results can be plausibly explained by reduced volume of the duplicate samples (less than half the original sample volume), meaning less extracted RNA in the duplicate assay. In addition, there was potential nucleic acid degradation as the duplicate samples had been stored for a considerably longer period than the original samples. The qPCR findings also suggest the possible presence of the virus in additional sympatric taxa (*M. australis, Cynopterus brachyotis and Chaerephon plicata*) and in additional locations (Puning Cave), but limits of detection issues and small sample volumes again precluded corroboration and sequencing. Limited blood volumes also constrained the use of additional assays such as cell culture and next generation sequencing. For context, around 85 % of the bats we screened weighed less than 100 g. In combination with our ethical decision not to destructively sample the bats, this meant that individual blood volumes were frequently much less than 100 μl.

The serologic findings in flying-foxes, in conjunction with the molecular findings in insectivorous bats, suggest that ebolavirus infection is taxonomically widespread in Philippine bats. Also, while ebolaviruses have previously been detected in other *Pteropodidae*, this is the first reported detection in flying-foxes. The stronger serologic response of one sample to EBOV than RESTV antigen in the Western blot is intriguing, and parallels recent findings from *Rousettus* fruit bats in Asia [10]. While acknowledging the potential for non-specific binding in the recombinant N protein-based Western blot, and for cross-reactivity with heterologous antigens [16], the findings could suggest that more than one strain of ebolavirus is circulating in the source population. All three Western blot corroborated seropositives were *A. jubatus*, and all were captured at the same roost, which is periodically shared with *P. vampyrus*. The uncorroborated ELISA-positive bat was a captive *P. vampyrus* from a different location. This scenario supports the veracity of the serologic findings. Additional samples are needed to further interpret the findings. The absence of positive serology in *M. schreibersii* given the positive PCR findings warrants discussion. In an endemic infection scenario, positive serology would expected in the source population from which viral RNA was detected. However, in a scenario of recent introduction of infection to a population, limited seroconversion in the presence of infected individuals would not be unexpected. The lack of sequence variation in all three PCR-positive *M. schreibersii* is consistent with the latter.

Our findings of RESTV infection in Philippine bats are supported by those of Taniguchi et al. [17]. They reported antibodies to RESTV in *Rousettus amplexicaudatus* from two locations in Luzon. As they sampled different bat populations, and one to two years prior to our study, our negative findings in *R. amplexicaudatus* in this study, while frustrating, are not overly surprising given the cryptic nature of filovirus infection and detection in bats [5]. Indeed, Tanaguchi et al. [17] screened 141 bats in total from 17 species, only confirmed RESTV-specific antibodies in 3 of 16 *R. amplexicaudatus*, and failed to detect any RESTV-specific amplicons by RT-PCR.

The decision to pool samples in the initial screening PCR reflected logistical constraints, however any saving in cost and time is countered by a loss of diagnostic sensitivity, which becomes particularly problematic when modest amounts of genetic material are present in the samples. In addition, the low level Ebola viral RNA detected from non-invasive swabs has prompted some studies to use tissue samples to maximise the probability of detection in infected bats (e.g., Amman et al. [8]). However, in this study we were constrained from destructively sampling bats, and thus our scope for viral detection may have been reduced. The aim of the study was to identify presence or absence of infection in bat taxa, and an optimistic target sample size was set to allow robust epidemiological interpretation of negative findings. This sample size was not met for any species or genus, and accordingly we refrain from making any interpretation on the lack of detection in any taxa. Conversely, our detection of infection in the modest sample of *M. schreibersii* indicates that, at the time of the study, infection prevalence was substantially higher than our conservative design prevalence.

## Conclusion

We found both molecular and serologic evidence of RESTV infection in multiple bat species in the Philippines. RESTV RNA was detected by quantitative PCR in oropharangeal swabs taken from *Miniopterus schreibersii*, with three samples yielding a product on hemi-nested PCR whose sequence had a single nucleotide difference from sequence of the pig isolate in Bulacan province. Further, uncorroborated qPCR detections may indicate RESTV nucleic acid in *M. australis*, *C. brachyotis* and *Ch. plicata.* In addition, we detected three seropositve *A. jubatus* using both Western blot and ELISA, suggesting that ebolavirus infection is taxonomically widespread in Philippine bats. However, given the evident low prevalence and low viral load of RESTV in bats, expanded surveillance in future studies is needed to elaborate our findings, and more broadly to elaborate the taxonomic and geographic occurrence of ebolaviruses in bats in the region. The recent detection of RESTV in pigs in China [18] highlights the need for the ecology of this virus to be better understood.

## Methods

### Study locations

Fieldwork was undertaken at two locations on the Philippine island of Luzon: Bulacan Province (13–26 April, 2010) and Subic Bay Freeport Zone (20 Nov-7 Dec, 2010) (Fig. 1). Bulacan Province was the focus of RESTV detections in pigs and associated pig workers, and the focus of our initial surveillance. The primary field locations in Bulacan Province were Biak na Bato National Park in the municipality of San Miguel (N 15° 06’ 33.9” E 121° 05’ 44.6”) and Puning Cave in the municipality of Doña Remedios Trinidad (N 14° 57’ 29.7” E 121° 05’ 27.4”). Biak-na-Bato National Park is an extensive protected area comprising forested riverine gorges and cave networks. Puning Cave is a riverine limestone cave complex within remnant forest habitat, surrounded by farmland. Both locations have diverse and abundant bat populations. A known flying-fox roost in the Cubi area of Subic Bay Freeport Zone (N 14° 47’ 16.63” E 120° 16’ 22.02”) was the focus of the later surveillance [19]. The roost is in a peri-urban forest remnant within an urban and farmland mosaic adjacent to an extensive area of largely intact forest.

### Bat capture and sampling

In Bulacan Province, sampling targeted insectivorous bats and small fruit bats, including taxa (or related taxa) previously associated with filoviruses in Africa [1]. We strategically deployed mist-nets and harp traps [20] after dusk to capture bats as they exited caves or foraged through the evening. Nets were continuously monitored, and bats removed on capture and placed in individual cotton bags; harp traps were monitored either continuously or periodically, the latter typically at hourly intervals, and bats removed from the holding bag of the trap and placed in individual calico bags. Bags were carried to the processing station (maximum 10 min) and hung from horizontal lines at minimum of 150 mm apart to ensure adequate ventilation. Following sample and data collection, bats were offered fruit juice for hydration and energy, and immediately released.

At Subic Bay, the sampling targeted pteropodid fruit bats (flying foxes), which were captured by mist-net [20] pre-dawn and post-dusk in the immediate vicinity of the known roost. Captured bats were held individually in cotton pillowcases and transported 3.4 km by vehicle to the *Wildlife in Need* Rescue Centre for processing. Bats were sequentially anaesthetised using the inhalation agent isofluorane [21]. Following data and sample collection, bats were recovered from anaesthesia, offered fruit juice (for hydration and energy), and released at their capture location within four hours of capture [19].

### Sample and data collection

Biological samples were collected from bats using non-lethal, minimally invasive techniques by multiple teams, each including a veterinarian and wildlife biologist with experience in handling bats. In turn, each bat was removed from its bag, and species, sex, pregnancy/lactation status, forearm length, weight and body condition score recorded [20]. A venous blood sample was collected as described by Smith et al. [22] for small species (<100 g), and by Epstein and Field [20] for flying-foxes (>500 g), and stored at 4C on wet ice in the field. Blood sample volume did not exceed 1 % bodyweight in accordance with animal ethics guidelines [23]. Duplicate oropharyngeal, urine/urogenital and rectal swabs were collected where possible, placed in lysis buffer (Nuclisens, Biomerieux, USA), and temporarily stored in a dry shipper at −70C. Samples were transported to the Bureau of Animal Industries (BAI) Philippine Animal Health Centre (PAHC) laboratory in Quezon City daily. Serum was yielded from the blood samples by centrifugation, and all samples collated into two sets where possible, one of which was archived at the PAHC laboratory, and the other subsequently forwarded to the CSIRO Australian Animal Health Laboratory (AAHL) in Geelong, Australia. Cross-contamination in the field was avoided by holding each bat in a separate clean bag from capture, processing individual bats sequentially, and adopting appropriate biosecurity protocols such as changing or disinfecting gloves between bats, disinfecting the immediate worksite and non-disposable equipment between bats, and using disposable and sterile consumables.

### Laboratory analyses

Serology and molecular assays were undertaken at AAHL. Samples were handled at BSL 4 until inactivated. An indirect ELISA (using mixed recombinant RESTV and EBOV (formally ZEBOV) N antigens) was used to screen sera, with Western blot (using individually run recombinant RESTV and EBOV N antigens) performed on ELISA-positive sera as described in Olival et al. [10]. Cut-off values to determine ELISA-positive sera were determined using a statistical approach as described in Pourrut et al. [1] and Olival and Hayman [5]. Confirmatory Western blot analysis was conducted as described in Hayman et al. [24]. Molecular assays comprised quantitative (q) and conventional RT-PCR in series. Swabs of the same sample type were pooled (five per pool) and RNA extraction undertaken using a QIAamp viral mini kit according to manufacturer’s instructions. Eluates were tested using a US CDC qPCR which targeted the RESTV NP gene (P. Rollin, 2010, pers. comm.). A sample yielding a repeatable C_t_ value of less than 40 was regarded as positive, and the authenticity of the amplified products corroborated by melt curve analysis; a sample yielding a repeatable C_t_ value of 40–45, or a non-repeatable C_t_ value of less than 40 was regarded as potentially positive. All other samples were regarded as ‘not detected’. Positive and potentially positive pools were re-tested in the same assay, as were the component individual samples. Where adequate sample remained, positive or potentially positive individual samples were tested by a PCR targeting the NP gene [25] adapted to a hemi-nested PCR with a second forward primer (FiloNP-hnFe – TGATGGTAATCTTYAGATTGATGAGG) in an attempt to gain adequate product for direct sequencing. Purified PCR products were sequenced at the AAHL sequencing facility using a BigDye Terminator v1.0 Kit (Applied Biosystems) and an ABI PRISM 377 DNA Sequencer (Applied Biosystems). Every nucleotide was sequenced with a minimum of threefold redundancy to ensure a consensus and repeatable sequence data. The Clone Manager and Align Plus programs in the Sci Ed Central software package (Scientific and Educational Software) were used for sequence management and analysis. Phylogenetic analysis based on the 519 bp fragment of the NP genes from different Ebola virus sequences was conducted using the MEGA5 program [26]. The phylogenetic tree was constructed using the maximum likelihood algorithm with bootstrap values determined by 1,000 replicates.

### Statistics and data analysis

The study employed a cross-sectional design to investigate the ‘presence or absence’ of RESTV infection in Philippine bats. A target sample size of 120 individuals per taxa was set, based on the averaged ZEBOV and MARV seroprevalence reported in Pourrut et al. [1], to allow negative findings to be interpreted as providing 95 % statistical confidence of absence of infection in the taxa at a design prevalence of 2.5 %. The findings are presented as descriptive summary statistics.

### Animal ethics

Fieldwork was carried out under the Philippine Government *Protected Areas and Wildlife Bureau* (now Biodiversity Management Bureau) permit no. 2010–197, issued following approval from the *Philippines National Wildlife Management Committee*. The latter is responsible for assessing applications for the use of wildlife for scientific research in accordance with the Philippines *Rules and Regulations on the Conduct of Scientific Procedures Using Animals*. The permit approved the defined study procedures for capture, handling, sample collection, and release of bats, including *Acerodon jubatus,* listed as ‘Endangered’ by the *International Union for Conservation of Nature* (IUCN) and the Philippine *National List of Threatened Wild Fauna*. All procedures reflect current best practice, minimising stress and discomfort, and reducing the risk of injury, mortality, and interference to natural behaviour. Local approvals were obtained from the Protected Area Management Board of Biak Na Bato National Park, the Municipality of Doña Remedios Trinidad, and Subic Bay Metropolitan Authority.
